# Low grade malignant eccrine spiradenoma of the vulva: case report, review of the literature and discussion about the role of p53 and HPV

**DOI:** 10.1186/s13000-020-00945-9

**Published:** 2020-03-14

**Authors:** Xavier Catteau, Nicky D’haene, Jean-Christophe Noël

**Affiliations:** 1 CUREPATH (Chirec institute- Brussels, CHU Tivoli – La Louvière), Rue de Borfilet, 12A, 6040 Jumet, Charleroi, Belgium; 2 Pathology Department, Erasme University Hospital, Université Libre de Bruxelles, CP 610, Route de Lennik, 808, 1070 Brussels, Belgium

**Keywords:** Eccrine, Spiradenoma, Malignant, Vulva, HPV, p53, Skin tumor, Case report, Rare, Review, Low grade, TP53, Mutation, Sequencing

## Abstract

**Background:**

Malignant eccrine spiradenoma is one of the rarest sweat-gland tumors. Here, we describe a rare case of low grade malignant eccrine spiradenoma located at the vulva.

**Case presentation:**

The vulvar lesion was described as a mass measured 3.5 cm and located in the dermis and subcutis with no attachment to the epidermis. The neoplasm was arranged in ragged sheets or solid nodules sometimes with focal necrosis. The tumor cells had hyperchromatism, pleomorphism, and prominent nucleoli with high mitotic index and KI-67 estimated at 70–80%.

**Conclusions:**

It’s only the fifth case of malignant eccrine spiradenoma localized at the vulva. This is the first time that an HPV genotyping was made in this type of lesion with no HPV found while the p16 expression was diffuse. Moreover, it’s the first time that a p53 mutation is detected by sequencing in this location.

## Background

Malignant eccrine spiradenoma (MES) is one of the rarest sweat-gland tumors, firstly reported in 1972 by Dabska [1] that can occur de novo or more commonly arises on a preexisting often long-standing eccrine spiradenoma [2]. The latency period before malignant transformation ranges from 6 months to 70 years. To date, about 120 cases have been reported, including 4 in the vulva [3–5]. Here, we describe a rare case of low grade MES located at the vulva not associated with Human Papilloma Virus (HPV) but with a p53 mutation.

## Case presentation

**Fig. 1:**
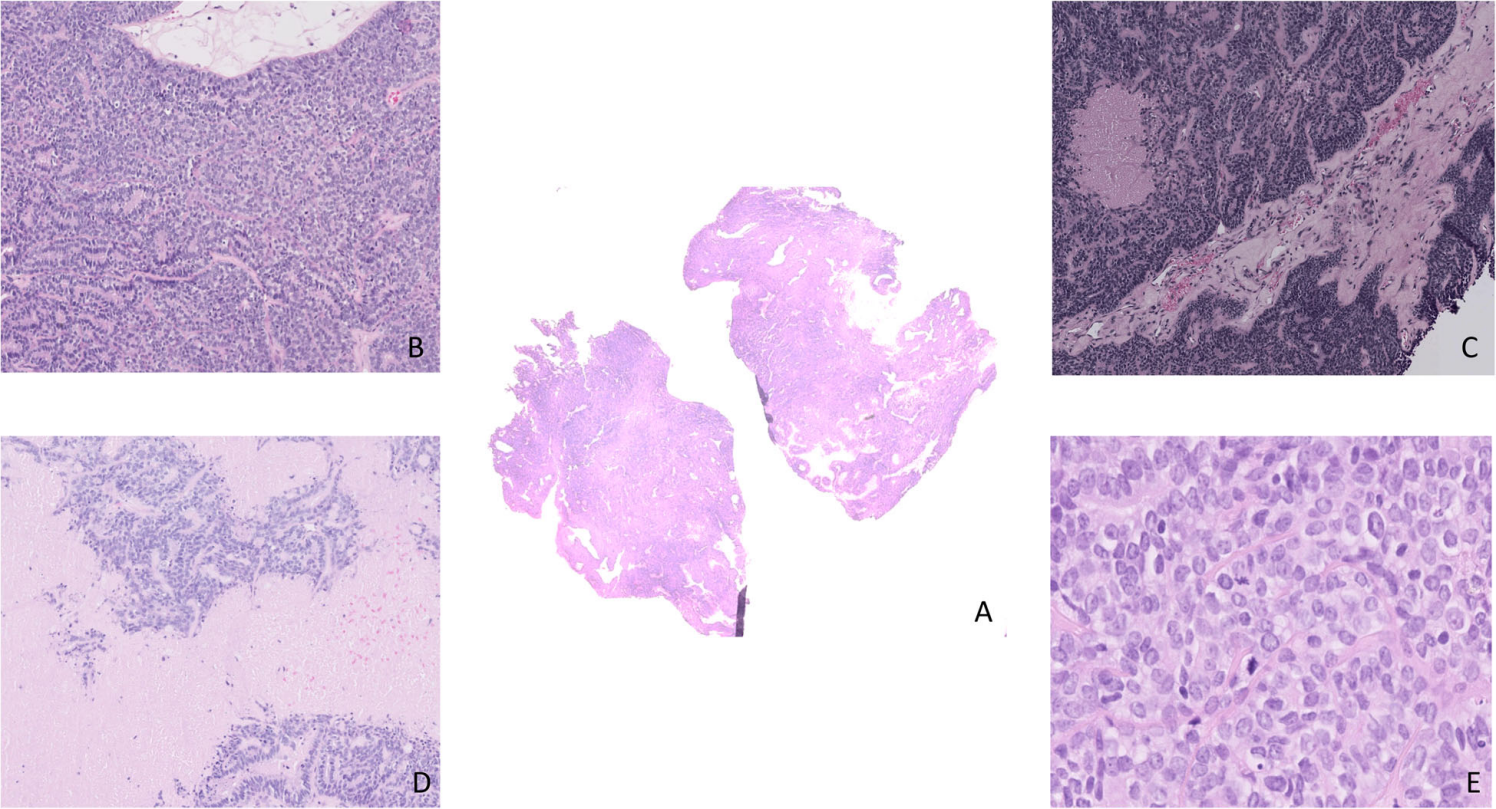
**a**. Tumor aspect at low power × 20. **b**. Tumor arranged in solid aggregates and cords of basaloid tumor cells × 40. **c**. Fibrous and vascularized tumoral stroma with a slight lymphocytic infiltrate × 100. **d**. necrosis dissectinf the tumor × 40. **e**. Basaloïd tumor cells with mild to moderate nuclear atypia with numerous and atypical mitotic figures

**Fig. 2:**
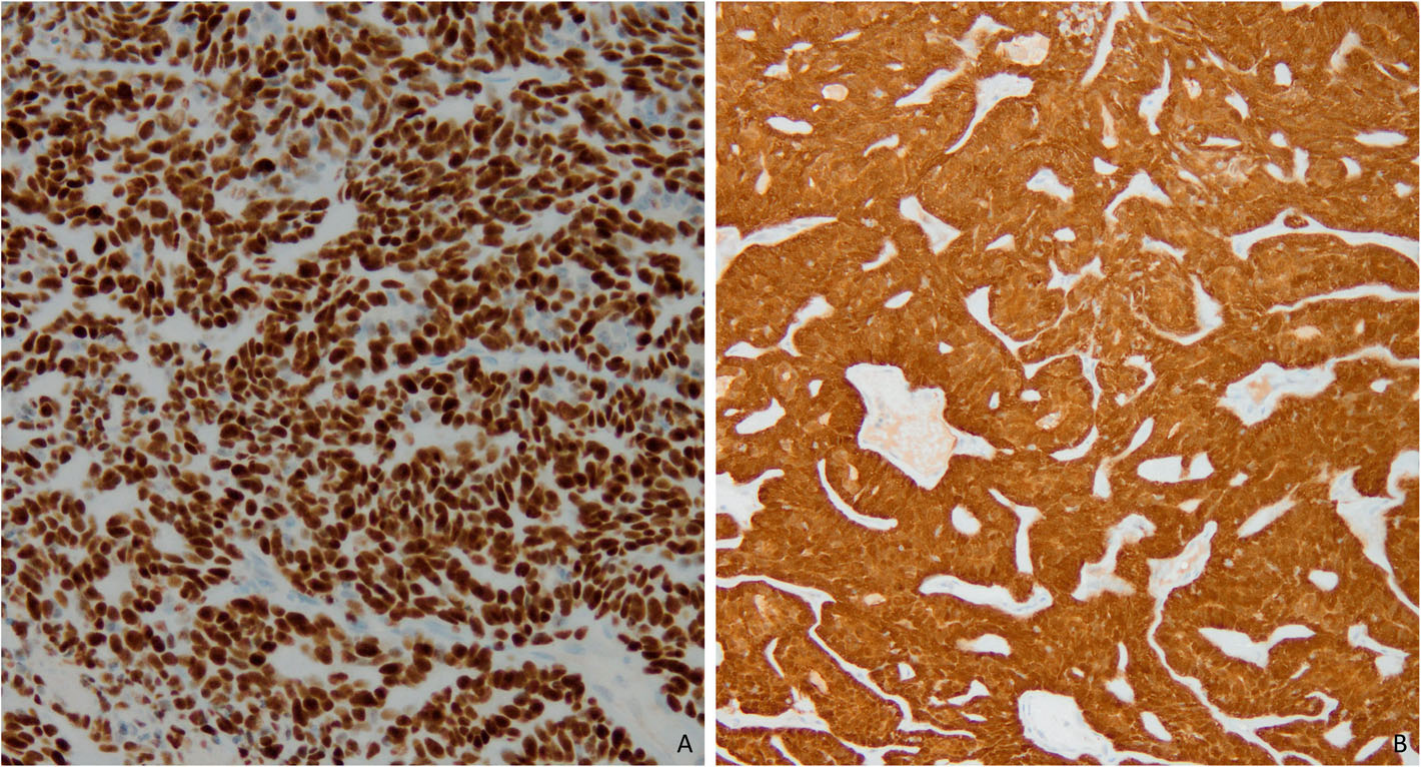
**a**. Strong nuclear expression of p53 mutation in tumor cells with mutation of TP53 gene by NGS. **b**. Strong nuclear and cytoplasmic expression of p16 protein in tumor cells with no association with HPV

This is a fifty-four-year old woman. In the past (2000), she had an invasive breast carcinoma treated by breast conserving surgery, chemotherapy and radiotherapy. She was perimenopausal and had no treatment. She presented a painless cystic mass on the right minor labia of the vulva. The minor labia skin was remarkable for a relatively well-defined cystic blue mass. The mass measured 3.5 cm. Histological examination showed a tumor located in the dermis and subcutis with no attachment to the epidermis. The lesion was consistent with a malignant adnexal neoplasm arranged in ragged sheets or solid nodules sometimes with focal necrosis. The tumor cells had hyperchromatism, pleomorphism, prominent nucleoli and moderate amounts of amphophilic cytoplasm. The mitotic activity was high with a mitotic index count estimated at 37 mitosis per 10 high power field and some of them were atypical. The KI-67 index was estimated at 70–80%. The intervening stroma is fibrous and vascularized with a slight lymphocytic infiltrate. Neither vascular invasion nor perineural invasion was identified [Fig. 1]. The tumor was positive for cytokeratin-7, CD117, carcinoembryonic antigen (CEA) (focal), GATA-3 and p16 (diffusely) and p53 (diffusely) [Fig. 2]. On the other hand, there was no expression of androgen, estrogen, progesterone receptor, Ber-EP4, Cerb-2/Neu, chromogranin, CD56 and GCDFP-15. As the tumor expressed diffusely the p16 protein, we extracted DNA to make HPV genotyping, but this test was negative (BD Onclarity™ HPV test with BD Viper LT). We also made a sequencing (cancer panel) of the lesion by Next-Generation-Sequencing (NGS) (Ion Torrent/PGM sequencing) and we found several mutations of the p53 gene (p.R158H, p.R175H, p.N2391, p.R273H, p.R280G).

## Discussion and conclusions

Malignant sweat gland tumors are rare accounting for 0.005% of all skin tumors. It tends to preferentially involve trunk and extremities [6]. Direct transition from benign to malignant neoplasm was noted in approximately half of cases [7, 8]. In this category of neoplasms, one hundred twenty cases of MES have been published and were most often localized on the ventral aspect of the upper trunk. Median patient age was 60 years (range 8–92 years) [9, 10]. To the best of our knowledge, it’s only the fifth case of MES localized at the vulva [11–14] and this is the fisrts time that HPV genotyping was made with no HPV found. Moreover, it’s the first time that a p53 mutation is detected by sequencing in this location. As a result of the rarity of malignant spiradenoma, the correct diagnosis of this tumor entity is a challenge, particularly when it occurs in a rare location such as the vulva. The typical manifestation of MES is a chronic (long-standing preexisting lesion) reddish-blue skin mass with recent rapid growth, which may show erythema, tenderness, ulceration, pain and bleeding [2, 3, 5]. The malignant features are not specific and include solid aggregates and cords of basaloid tumor cells with mild to moderate nuclear atypia, loss of two cell populations, an infiltrative pattern of growth, numerous and atypical mitotic figures, necrosis, lymphovascular and perineural invasion [3]. Mitotic rates vary between 4 and 32 per 10 high power fields (× 40) counted (average 17.2) [4, 11]. All of these criteria are not met in all MES. Moreover, two distinct morphological patterns of MES have been described, the most common form consisting of an obviously malignant pleomorphic tumor (high-grade carcinoma) and a second low-grade form that closely mimics benign spiradenoma [3]. The tumor cells may express cytokeratins (CAM 5.2, CK7), epithelial membrane antigen (EMA), p53, S100, CEA and sometimes estrogen and progesterone receptor (presumably because of the common embryologic origin of eccrine sweat glands and mammary gland tissue) [3, 5, 15–17].

To the best of our knowledge, p16 expression is not described in spiradenoma. Moreover, we didn’t find the research of HPV by genotyping in the literature for this type of lesion. On the basis of these analysis, HPV is not associated with genital spiradenoma. Differential diagnosis should include others adnexal neoplasms such as cylindroma, malignant nodular hidradenoma, and basal cell carcinoma. Anaplastic carcinoma, adenocarcinoma, squamous cell carcinoma, and carcinosarcoma should also be considered [3]. It should be noted that one case of vulvar carcinosarcoma developed from ex-eccrine spiradenoma has been described by Baker et al. [12]. Because of its rarity, the optimal treatment for MES is unclear [5] and its biologic behavior is uncertain [4, 6]. Few authors added magnetic resonance imaging (MRI) for defining tumor extent and FDG PET/CT for detection of metastases, especially if clinical findings were unremarkable. The mainstay of therapy is surgical excision, which may be curative in some cases [5]. Although there is no established treatment regimen, wide resection with 1-cm, tumor-free, circumferential and deep margins down to the fascia has been advocated [18]. Regional lymph node excision has been recommended for clinical suspicious tumor positive regional lymph nodes [2, 6] and sentinel node biopsy is considered useful in clinically unsuspicious ones [2]. Some data show fewer recurrences and deaths after tumor resection with tumor free margins compared with unresected lesion (recurrence 23% vs. 43%), (death 8% vs. 43%) [5]. No definitive statement can be made regarding adjuvant therapy in patients without distant disease [2]. It is generally known that sweat gland tumors are radioresistant [19]. Due to the rarity of these lesions, the experience with chemotherapy is limited [20]. Tamoxifen, a selective estrogen receptor modulator, has been tried as an adjuvant therapy in a case of estrogen receptor positive MES [18]. Follow-up should include recurrent imaging every 3 months at the beginning [5, 6, 11]. Recurrences are reported in 17.5 to 57% of cases and metastases in lymph node or in lungs, brain, and liver are observed in 40% of cases after a mean follow-up period of 35.23 months. 17.5% of patients develop distant metastases and die of disease after an average time period of 11 months following diagnosis [6, 21, 22]. The presence of lymph node or distant metastasis of MES significantly reduced survival [2]. In a study of 36 cases of non-metastatic disease, the 35 patients treated with local surgical resection alone yielded a disease-free survival rate of 100% with a mean follow-up period of 33 months. In the case of distant metastasis, the median survival time was longer for patients treated with adjuvant therapy than with local resection alone, but this outcome was not statistically significant [2]. It appears that tumors with sarcomatous component, not found in our patient, have a more aggressive behavior than those without such features [11].

p53 is a tumor suppressor protein encoded in humans by the TP53 gene. It is a crucial component in multicellular organisms, as it regulates the cell cycle and helps prevent cancer and is the most frequently altered gene in human cancers. As an anti-cancer promotion agent, it can activate DNA repair proteins when DNA has sustained damage, induce growth arrest by holding the cell cycle at the G1/S regulation point on DNA damage recognition, or initiate apoptosis if DNA damage proves to be irreparable [23]. Overexpression of tumor protein p53 was found positive in 90% in a study of 31 MES [5] and mutations of p53 gene is a known indicator for malignant transformation [24]. The present case corresponds to a low grade malignant spiradenoma but with mutations of p53. We didn’t find a clear difference between the high- and low-grade MES concerning the status of p53 mutation in the literature. In our case, we described for the first time several mutations of p53 gene in a low-grade subtype localized at the vulva. The staining may be focal and weak, particularly in low-grade carcinomas [25]. Nevertheless, Biernat found no p53 immunopositivity among benign spiradenomas, whereas all malignant spiradenomas demonstrated p53 overexpression [24]. Lee et al., in their case of malignant spiradenoma found no expression of p53 in the benign areas; in the malignant areas only “occasional nuclear reaction was disclosed” [26]. It’s generally thought that significant p53 immunopositivity reflects the presence of the mutant forms of the gene. However, in a study of several cases of MES only one revealed a p53 mutation [27]. There are several possible explanations for this result. It could be due to tumor heterogeneity and the presence of a mutation in only a minority of tumor cells below the analytical sensitivity of the molecular assays employed. Another explanation is that there may be deregulated expression of p53 protein due to redundancy of positive, or lack of negative regulators of p53, for example, p14ARF and MDM2 [27]. Finally, Single Nucleotide Polymorphism (SNPs) might influence the expression and stability of p53 protein [25]. No data was found in the literature about the expression of p16 in MES. In our case, we found a strong nuclear and cytoplasmic expression of p16. Due to the location of the tumor (vulva), we wondered if this expression was related to the presence of HPV infection. We didn’t find HPV infection, but we know that p16 is not only associated with HPV infection but it’s also a tumor suppressor gene implicated in several tumors [28].

In conclusion, we have described for the fifth time a case of a localized MES at the vulva and we have identified for the first time several mutations of the p53 gene and an absence of HPV in a low-grade subtype at this location.

## Data Availability

Not applicable.

## Abbreviations

CEA: Carcinoembryonic Antigen
EMA: Epithelial Membrane Antigen (EMA)
HPV: Human Papilloma Virus
MES: Malignant Eccrine Spiradenoma
MRI: Magnetic Resonance Imaging
NGS: Next-Generation-Sequencing
